# Tapping into 5-HT_3_ Receptors to Modify Metabolic and Immune Responses

**DOI:** 10.3390/ijms222111910

**Published:** 2021-11-02

**Authors:** Helen Irving, Ilona Turek, Christine Kettle, Nor Yaakob

**Affiliations:** 1 Department of Pharmacy and Biomedical Sciences, La Trobe Institute for Molecular Science, La Trobe University, Bendigo, VIC 3550, Australia; i.turek@latrobe.edu.au (I.T.); c.flens@latrobe.edu.au (C.K.); 2 Drug and Herbal Research Centre, Faculty of Pharmacy, Universiti Kebangsaan Malaysia, Jalan Raja Muda Abdul Aziz, Kuala Lumpur 50300, Malaysia; nsy@ukm.edu.my

**Keywords:** serotonin receptors, 5-hydroxytryptamine receptors, metabolism, adipose tissue, chemotherapy induced vomiting and emesis (CINV), inflammation, HTR3 variant associations, HTR3 single nucleotide polymorphism, 5-HT_3_ receptor-based therapies

## Abstract

5-hydroxytryptamine type 3 (5-HT_3_) receptors are ligand gated ion channels, which clearly distinguish their mode of action from the other G-protein coupled 5-HT or serotonin receptors. 5-HT_3_ receptors are well established targets for emesis and gastrointestinal mobility and are used as adjunct targets in treating schizophrenia. However, the distribution of these receptors is wider than the nervous system and there is potential that these additional sites can be targeted to modulate inflammatory and/or metabolic conditions. Recent progress in structural biology and pharmacology of 5-HT_3_ receptors have provided profound insights into mechanisms of their action. These advances, combined with insights into clinical relevance of mutations in genes encoding 5-HT_3_ subunits and increasing understanding of their implications in patient’s predisposition to diseases and response to the treatment, open new avenues for personalized precision medicine. In this review, we recap on the current status of 5-HT_3_ receptor-based therapies using a biochemical and physiological perspective. We assess the potential for targeting 5-HT_3_ receptors in conditions involving metabolic or inflammatory disorders based on recent findings, underscoring the challenges and limitations of this approach.

## 1. Introduction

Isolation of serotonin (sero = serum and tonin = vasoconstrictive) by Rapport [1] and the confirmation that the compound “enteramine” isolated by Erspamer was the same compound and actually 5-hydroxytryptamine (5-HT) [2] opened up the field of serotonin research. Initially two different types of 5-HT receptors were characterized as D and M receptors in guinea pig ileum, where D refers to receptors blocked by dibenzyline and M refers to those blocked by morphine found mainly in muscle and nervous tissue, respectively [3]. Radioligand binding studies, over the next 20 years or so, revealed that the situation was more complex and that further subtypes of 5-HT receptors existed. During the mid-1980s, 5-HT receptors were classified into three categories, 5-HT_1_ receptors containing some D receptors and 5-HT_1_ binding sites, 5-HT_2_ receptors mainly D receptors and 5-HT_3_ receptors being equivalent to M receptors [4] and then the 5-HT_3_ receptor was found to be an ion channel [5].

**Figure 1:**
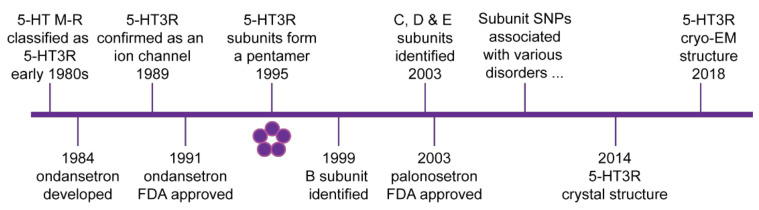
Schematic timeline of major discoveries in 5-HT_3_ receptor biology. 5-HT_3_ receptors (5-HT3R) are cysteine loop ligand gated ion channels formed by a pentamer arrangement of subunits as shown by the five circles. Initial clinical approval by the US Food and Drug Administration (FDA) of the first generation 5-HT_3_ receptor antagonist ondansetron and the second-generation antagonist palonosetron are also indicated. From the early 2000s, several single nucleotide polymorphisms (SNPs) and other polymorphisms have been associated with various clinical disorders. Further details are outlined in the text.

Now it is known that there are seven classes of 5-HT receptors and that M or 5-HT_3_ receptor are unique as ion channels and are distinct from the others, which are G protein coupled receptors [6,7,8]. The 5-HT_3_ receptor is a member of the cysteine loop ligand gated ion channel family. The receptor is arranged as an assembly of five subunits around a central pore [9] and carries cations (e.g., sodium, potassium, and calcium ions) into cells [5]. More recent studies have revealed that several different 5-HT_3_ receptor subunits (A, B, C, D and E) are expressed in humans [10,11,12,13]. Since the differential subunit composition of a 5-HT_3_ receptor can alter its properties, we refer to the 5-HT_3_ receptor of undefined subunit permutation as 5-HT_3_ receptors for simplicity. However, the presence of the A subunit is essential for the formation of functional plasma membrane ion channels [10,14], and only 5-HT_3_A subunits can form a functional homopentamer. A schematic outline of several major events in the study of 5-HT_3_ receptors is shown in Figure 1. Here, we first briefly review 5-HT_3_ receptor distribution, function and 5-HT_3_ receptor antagonists currently used in the clinic. The focus of this review is to discuss how recent structural insights combined with enhanced understanding of the role of 5-HT_3_ receptors, contribute to potential novel therapeutic directions in inflammation and metabolic disorders.

## 2. Distribution of 5-HT_3_ Receptors

Since this is a well-documented and reviewed area (e.g., [8,15,16,17,18,19,20,21,22]), we will briefly describe the distribution of 5-HT_3_ receptors throughout the body focusing on known and potential target regions and the potential contribution of the different subunits. As only the A subunit was initially identified, earlier studies merely report its expression. Discovery of the different subunits revealed their distribution across human body organ systems [11,13,23]. Some selective subunit localization is evident in humans and other species. Most notably, rodents only contain A and B subunits [14] and differ in other aspects of their cellular function and physiology to humans [24,25,26].

Autoradiography mapping studies using radiolabeled 5-HT_3_ receptor antagonists such as granisetron revealed high density of 5-HT_3_ receptors in human brainstem including nucleus tractus solitarius, area postrema, and dorsal motor nucleus of the vagus nerve [27,28,29]. All these areas are important for starting and coordinating the vomiting reflex and are thus likely to contribute to the anti-emetic effects of 5-HT_3_ receptor antagonists [15,30]. 5-HT_3_ receptors also occur in the medulla oblongata, hippocampus, caudate and putamen [31,32,33]. Heteromeric expression of 5-HT_3_ receptors [34,35] is likely throughout the human brain correlating with the widespread distribution of transcripts of the different subunits reported in brain regions [13,14]. Postsynaptic 5-HT_3_ receptors modulate fast excitatory synaptic transmission when activated while the presynaptic 5-HT_3_ receptors modulate neurotransmitter release indicating an interconnected and complex network [15] that, at least in the mouse model, modulates cerebellum synaptic plasticity [36] in cellular events involved in cortical construction [37]. Distribution patterns of 5-HT_3_ receptors have encouraged the consideration of 5-HT_3_ receptor antagonists generally as adjunct therapies in treating various neurological and psychiatric disorders [18,38]. Of note, mutations in genes encoding different 5-HT_3_ subunits have been associated with the occurrence of bipolar affective disorder, depression, and schizophrenia, and may be of clinical relevance in predicting the effectiveness of their treatment (Table 1).

5-HT_3_ receptor subunit expression occurs in the enteric nervous system and other regions of the gut, and they are particularly known for their involvement in brain-gut circuitry. Gene transcripts encoding 5-HT_3_ receptor subunits have been detected in the human colon [13,14,105,106,107,108] and the stomach [109]. Unexpectedly, there was a spatial differential in the tissue distribution of the E subunit being only present in the mucosal layers [108] and a single nucleotide polymorphism in the gene encoding E subunit is associated with diarrhea predominant irritable bowel syndrome [42]. Other polymorphisms in the gene encoding C subunit are also useful in predicting treatment response [97] (Table 1). Interestingly, the expression level of 5-HT_3_ receptors in intestinal mucosa of patients with diarrhea predominant irritable bowel syndrome is significantly higher than those of healthy subjects, thus 5-HT_3_ receptors may be involved in the pathogenesis of this disease and act as a potential target for intervention [97,110].

5-HT_3_ receptors in the gastrointestinal tract modulate physiological responses such as gastric emptying, colonic peristalsis and transit [111,112,113]. Thus, 5-HT_3_ receptor antagonists are used to treat diarrhea predominant irritable bowel syndrome [22,114,115] in addition to chemotherapy-induced nausea and vomiting (CINV) or post-operative vomiting (POV) [30,116,117]. Genetic polymorphisms in genes for 5-HT_3_B, 5-HT_3_C, and 5-HT_3_D, predominantly expressed in the gastrointestinal tract and various brain regions [13,14], may contribute to facilitating individual risk predictions (Table 1). 5-HT_3_ receptor expression has been detected in myenteric and submucosal plexus of human colon and rectum by autoradiography [118] and immunohistochemical [119,120] methods.

As this is discussed in detail in a later section, we only highlight here that 5-HT_3_ receptors are expressed in various cells of the immune system. Mainly, the 5-HT_3_ receptor A subunit has been reported in monocytes, chondrocytes, T-cells, B-cells, synovial tissue, platelets, dendritic cells [121,122,123,124,125,126,127] and is overexpressed in lymphomas [127]. The presence of 5-HT_3_ receptors is likely to reflect immune challenge as there is a significant increase of the A receptor subunit in peripheral blood mononuclear cells from asthmatic patients and those exposed to air pollution [128] leading to inflammatory responses that can potentially be down regulated with 5-HT_3_ receptor antagonists [121,124]. Similarly, the lack of or reduced expression of 5-HT_3_ receptors in psoriatic epidermis implies a role for these receptors in proliferation and differentiation of keratinocytes [129], one of the first lines of immune defense. Since 5-HT_3_ receptors are expressed in basal epidermal cells, particularly in acrosyringium and in the epithelium of hair follicles [130], they may be targeted for the treatment of chronic skin diseases and pruritus. 5-HT_3_ antagonists reduce the severity of serotonin-induced skin itch [131] and neuraxial opioid-induced pruritus [132]. Direct irradiation cell death evoked by serotonin in keratinocytes is mediated through 5-HT_3_ receptors, thus identifying these as a potential target for ameliorating ionizing radiation damage [133].

5-HT_3_ receptors are widely distributed in other human body organ systems highlighting the interconnectedness between physiological processes regulated via these receptors. 5-HT_3_ receptor subunits are found in respiratory, urogenital, renal, and cardiovascular systems [13,14,23]. It is highly likely that further interconnections will be established between systems involving those occurring between gastrointestinal regulation and emetic events [134,135,136], inflammatory bowel disease as well as pain and inflammation in various sites [8,16,17,18,135,137,138].

## 3. Structural Insights into 5-HT_3_ Receptor Action

**Figure 2:**
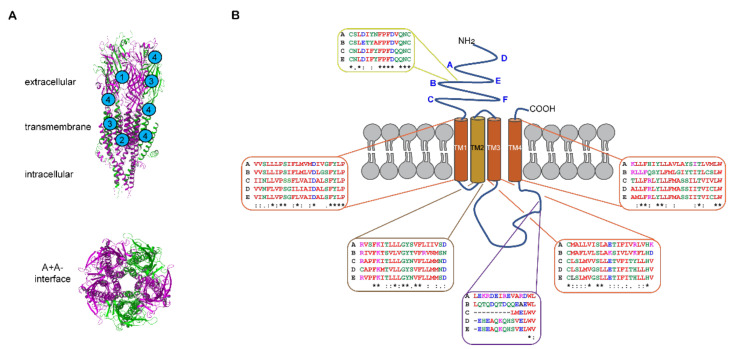
Architecture of 5-HT_3_ receptors. (**A**) Homology model of the human 5-HT_3_AC receptor assembled in the A_3_C_2_ stoichiometry showing membrane spanning side and central pore views (maroon = A and green = C subunits). Examples of proposed binding sites for competitive, non-competitive, and dual acting antagonists (labeled 1–3), and allosteric modulators (indicated with 4). The model is from [153] where A and C subunits were created from the mouse 5-HT_3_A receptor structure [139] as they have 95% and 58% homology with the mouse A subunit, respectively. (**B**) Schematic diagram of a single 5-HT_3_ receptor subunit showing the different domains and extracellular A–F loops (bolded in blue). A Clustal omega alignment was done on the human 5-HT_3_ receptor subunit protein sequences obtained from NCBI [154] and this is shown for the Cys-loop (yellow box), membrane-spanning α-helices of transmembrane domains 1 to 4 (TM1-TM4) (brown and orange boxes), the triple R region of the intracellular loop involved in pentamer assembly [155] and the low conductance associated with A homomer receptors [156] (purple box). Accession numbers: NP_000860 (A subunit), NP_006019 (B subunit), NP_570126 (C subunit), NP_001157118 (D subunit), and NP_872395 (E subunit). Identical residues are indicated with an asterisk (*), conserved residues with a colon (:), and semi-conserved residues with a full stop (.). Image drawn in Adobe Illustrator.

The structure of the mouse homologous 5-HT_3_A receptor revealed through crystallography [139] and cryo-electron micrography [140,141,142,143,144,145,146] provides considerable insight into 5-HT_3_ receptors and is reviewed [20,147]. The mouse A subunit shares 95% homology with the human A subunit, so these studies are readily translatable. The 5-HT_3_ receptor subunits each share a similar architecture and arrange in a pentamer with a central pore (Figure 2). Each subunit contains extracellular N- and C-termini, four transmembrane (TM) spanning α-helical-domains and an intracellular domain. The Cys-loop is located in the N-terminal region of the extracellular domain where cysteine and proline residues contribute to receptor conformation [148,149]. High-resolution structural studies have been particularly revealing about the extracellular and transmembrane domains of the homomeric receptor [140,141,142,143,144,145,146]. The extracellular N-terminal region contains the orthosteric ligand binding site that is created between two A subunits asymmetrically arranged to form the A+A- interface [141,142,143,144,145,146,147] (Figure 2A). The structural studies have, to a large degree, refined and elaborated on the molecular understanding of the orthosteric sites initially characterized through mutagenesis and ligand binding approaches [15,19,150,151]. The three-dimensional structures open up many opportunities to employ additional techniques such as molecular dynamics simulations [141,142,145,146] in conjunction with photo-crosslinking and mass spectrometry to delve further into the dynamics of ligand receptor interactions [152]. The transmembrane domains are arranged asymmetrically so transmembrane domain 2 (TM2) faces the pore and contributes most to ion interactions therein (Figure 2). The pore widens when 5-HT binds at the orthosteric site due to conformational changes twisting transmembrane domain 2 outwards [142,144,146,147].

The large intracellular loop between transmembrane domains 3 and 4 contains an intrinsically disordered domain refractory to structural studies. However, this intracellular domain is critically important in assembling 5-HT_3_A receptor pentamers [155,157]. This region has greater diversity in amino acid sequences between the different subunits (Figure 2B). The A subunit contains a triplet of arginine residues that are important in regulating conductance [156] and these residues form salt bridges that contribute to pentamer formation [155] possibly forming part of the explanation for the necessity of A subunits in heteromers. The intracellular domain is also necessary for direct interactions with acetylcholine receptor chaperone (RIC3) that enables post-translational trafficking to the plasma membrane [158,159,160,161]. A specific region of the intracellular domain in the A subunit directly interacts with RIC3 [158] but RIC3 binding sites have not yet been identified for the other subunits [161].

5-HT_3_ receptors are subject to post-translational modifications, including the disulfide bond in the Cys-loop present in all human 5-HT_3_ subunits, except 5-HT_3_D (Figure 2B), which impact their structure and function. These modifications include *N*-glycosylation in the extracellular N-terminus of human 5-HT_3_A and 5-HT_3_B subunits (at four and five asparagine residues, respectively) [162] important for cell surface trafficking and stability of the receptors [163,164,165]. Inhibiting glycosylation at any of the *N*-glycosylation sites reduced or prevented membrane expression of 5-HT_3_A subunits and interfered with the formation of 5-HT_3_ receptor binding site. *N*-glycosylation at N5 is less critical, probably due to its distance from the ligand binding loops (Figure 2B) than modification of N81, N147, and N163 and notably these human 5-HT_3_A subunit canonical motifs are conserved across species, likely reflecting their significance [163]. Site-directed mutagenesis studies confirmed that the disruption of any *N*-glycosylation site on human 5-HT_3_B subunit (N31, N75, N117, N147, and N182) resulted in reduced expression of the subunit in cell membranes, presumably incorporated within heteromeric 5-HT_3_ receptors [165]. Although *N*-glycosylation at N145, residing within the Cys-loop (Figure 2B), is conserved in both 5-HT_3_A and 5-HT_3_B subunits, and many other ligand-gated ion channels, it seems less important in facilitating 5-HT_3_B subunit membrane incorporation [165].

Phosphorylation is also a critical post-translational reversible protein modification. Phosphorylation of the large intracellular loop (Figure 2B) likely involves protein kinase A or C effects on ion conductance and rate of desensitization and depends on particular isoforms of 5-HT_3_A subunits present [166,167,168,169,170]. Effects of agents affecting the actin cytoskeleton together with point mutations of the potential phosphorylation sites suggested that protein kinase C modulates 5-HT_3_A receptor function and trafficking indirectly, likely via an F-actin-dependent mechanism [169]. Moreover, protein kinase CK2 enhances current flux through 5-HT_3_ receptor channels [171] while calcineurin—a calcium/calmodulin-dependent phosphatase—does not modulate the fast desensitization of 5-HT_3_ receptor, it may regulate channel activity via the modulation of the steady-state desensitization [172].

The lipid membrane itself is also involved with 5-HT_3_ receptors accumulating in lipid rafts—highly dynamic cell membrane microdomains enriched in cholesterol and glycosphingolipids [173]. The impairment of lipid rafts diminishes 5-HT_3_ receptor currents although only a minor proportion of the 5-HT_3_ receptors appeared to be constantly present in lipid rafts [174]. Interestingly, removal of cholesterol from the bilayer of large unilamellar vesicles containing homogenously distributed 5-HT_3_ receptors resulted in the formation of tightly packed patches [175]. Cholesterol binds to and regulates some ligand gated ion channels [176]. Molecular dynamics simulations have revealed sustained interaction between cholesterol and TM domain of 5-HT_3_A receptors, where the lipid penetrates a preactivated receptor monomer through binding between M1 and M4 helices, with hydrogen bonding between cholesterol and TM4 helix [148]. A lipid-binding pocket lined by TM3 of one subunit and TM1 and TM4 helices of the neighboring subunit, where R309 in TM3 and R435 in TM4 residues interact with the phospholipid has been revealed in 5-HT_3_ receptors in the absence of the ligand [140]. A further structural basis for the receptor modulation by lipids, together with a model for allosterically lipid-modulated asymmetric activation of the homopentameric 5-HT_3_ receptor reconstituted into saposin-based lipid bilayer discs, were recently proposed [146]. Apart from enabling the receptor to adopt an asymmetric conformation in the absence of the ligand, the lipid moieties were shown to facilitate a more compact, ‘coupled’ conformation of the receptor, with transmembrane domains compressed along the central axis, allowing free diffusion of sodium ions.

Arrangement of the 5-HT_3_ receptor heteromers has generated some controversy with earlier atomic force microscopy studies using antisera to tagged subunits suggesting that the AB heteromer had a B-B-A-B-A (A_2_B_3_) stoichiometry [177], whereas the consensus of multiple mutagenesis studies indicated that at least one A+A- interface is necessary in the 5-HT_3_AB receptors [151,178,179], favoring an A-A-B-B-A or A-A-B-A-B (A_3_B_2_) conformation. Confirmation of the A_3_B_2_ stoichiometry with separated B subunits was obtained using fluorescent proteins inserted into the intracellular loop between transmembrane domains 3 and 4 to obtain fluorescence intensity ratio (FIR) and FRET efficiency measurements [180]. The conformation of heteromers involving the other subunits is unknown but pharmacological studies involving agonist and antagonist binding strongly indicate that an A+A- interface is present like that which occurs with the AB heteromer [178]. Thus, 5-HT_3_A receptor homomers are likely to have more orthosteric binding sites than heteromers, possibly underscoring pharmacological differences in ligand binding between homomers and heteromers. Sequential ligand binding is proposed for A homomers especially in a lipid environment where cholesterol can contribute allosterically to receptor conformational changes [146,148].

## 4. Insights from Pharmacology and Electrophysiology Studies

Electrophysiological and pharmacological approaches have provided many insights into the action of the 5-HT_3_ receptor. The majority of studies use 5-HT_3_A or 5-HT_3_AB receptors as the C, D and E subunits in humans have been more recently discovered and are not expressed in all organisms [11,13,14]. 5-HT is the endogenous agonist and several synthetic 5-HT_3_ receptor agonists incorporating a basic amine, an aromatic ring and hydrogen bond acceptors are mainly used as experimental tools. The potent agonists commonly used in experiments include 2-methyl-5-HT, phenylbiguanide, and *meta*-chlorophenylbiguanide (m-CPBG) [8,181]. Differences in functional responses to agonists are evident between species [8]. An example involves the functional neural 5-HT_3_ receptors present along the length of the mouse, rat and guinea pig intestinal tracts that show differences in agonist efficacies between species and in separate regions of the tissue [182,183]. Interest has been generated in partial agonists as potential therapeutic agents and CSTI-300 is one such compound that has recently been characterized [184].

Electrophysiological studies reveal that at least part of the functional differences between species lie in the molecular structure and potential stoichiometry of the channel. 5-HT_3_ receptors mainly carry sodium and potassium ions following activation by 5-HT to mediate a rapidly activating and desensitizing inward rectifying current, although ion substitution experiments have revealed that 5-HT_3_ receptors also carry calcium ions [15]. 5-HT_3_ receptors are homomeric (all A subunits) or heteromeric (mixture of A and either B, C, D, or E subunits) ion channels [8,15,18,19,21,22]. There are variations in the number of subunits between species, as rodents do not express the C, D or E subunits unlike humans and many other species including cats, dogs, and cattle [14]. Therefore, most work has focused on the 5-HT_3_A or 5-HT_3_AB receptors [8]. Heterologous system studies have revealed several differences between 5-HT_3_A and 5-HT_3_AB receptors [185]. The marked difference in single channel conductance (5-HT_3_A is <1 pS versus 5-HT_3_AB at 16–30 pS) is due to the triplet arginine residues present in the portal region of the intracellular loop between the transmembrane domains 3 and 4 of the A subunit that are lacking in the B subunit [156]. The corresponding residues present in the C, D and E subunits differ from either the A or B subunits (Figure 2B). Few studies have been made with the C, D or E subunit and these subunits appear to contribute subtle changes to 5-HT or various agonists relative to the B subunit [14,161,186,187,188]. Distinctive albeit subtly different pharmacological profiles for 5-HT_3_AC, 5-HT_3_AD and 5-HT_3_AE receptors were revealed using partial agonists [187].

All known agonists and competitive antagonists bind at the orthosteric binding site in the extracellular N-terminal region, at the interface between two A subunits [15,19,141,142,143,145,151]. The competitive antagonists tend to be larger than agonists and are mainly represented by the setron family but include morphine and cocaine [3,8,18,22,151]. The first generation setrons including bemestron, tropisetron, granisetron and ondansetron have strong antiemetic activities and generally nanomolar affinity for 5-HT_3_A receptors [189,190,191,192,193]. Tropisetron also exhibits selective potent partial agonist activity at α7 nicotinic receptors not seen with other 5-HT_3_ receptor antagonists like ondansetron [194,195] while ondansetron at micromolar concentrations can attenuate solute carrier family 1 member 1 (SLC1A1 formerly known as excitatory amino acid transporter type 3 (EAAT3)) activity [196]. Ondansetron has more recently been shown to activate the ATP binding cassette subfamily A member 1 (ABCA1) activating transcription through the nuclear receptor subfamily 1 group H member 3 pathway (NR1H3) to stimulate apoliproprotein E (ApoE) secretion from astrocytes and liver cells [197]. Picrotoxin and related compounds, such as ginkgolides, block the 5-HT_3_ receptor channel [179,198]. Palonosetron is a second generation setron that has both competitive and non-competitive antagonistic properties [185,199]. Several ligands have allosteric properties acting at sites spatially distinct from the orthosteric ligand binding site and include anesthetics, n-alcohols, cannabinoids, steroids, as well as terpenes and pungent substances frequently occurring in plants [8,151,200,201,202,203]. Many of these positive or negative allosteric modulators bind to transmembrane sites of 5-HT_3_ receptors [202,204,205].

Therefore, the ligands can be classified by the site they bind: 1. competitive antagonists (e.g., ondansetron); 2. non-competitive antagonists (e.g., picrotoxin) acting via locations other than the orthosteric binding site; 3. dual acting antagonists (e.g., palonosetron [206,207]), which bind at orthosteric or transmembrane sites although structural studies indicate palonosetron only binds at the orthosteric site [145]; and 4. allosteric modulators that bind at sites distinct from the orthosteric site such as the interface of the extracellular and transmembrane domains [15,19,141,142,143,145,150,151] (Figure 2A). Importantly from an experimental and potentially clinical view (see below), ligands can have different potencies at 5-HT_3_A versus 5-HT_3_AB receptors [89,184,185,208,209,210]. For instance, the agonist m-CPBG can bind at all five interfaces of 5-HT_3_AB distinguishing it from 5-HT, which only binds at the orthosteric binding site at the A+A- interface within the 5-HT_3_AB receptor enabling allosteric modulation [208]. While VUF10166 has a high affinity for 5-HT_3_A receptors binding at the A+A- site, its affinity for 5-HT_3_AB receptors is lower and its effects are thought to be mediated by binding to a secondary A+B- site [211]. Various anticancer drugs inhibit or potentiate current in 5-HT_3_ receptors, and the degree differs with topotecan, irinotecan and imatinib depending on whether it is a receptor homomer of A subunits or heteromer of A and B subunits [89,212]. Whole-cell patch clamp recordings revealed that the dual acting antagonist palonosetron distinguishes 5-HT_3_ACE receptors from 5-HT_3_A, 5-HT_3_AC and 5-HT_3_AE receptors [188]. Most studies using C, D or E subunits use diheteromeric receptors in heterologous systems generally showing that these subunits only contribute subtle changes to agonists or antagonists compared to homomeric 5-HT_3_A receptors [14,153,161,186,187,188].

Since the discovery of additional 5-HT_3_ receptor subunits and various clinically relevant polymorphisms such as the single nucleotide polymorphism in the B subunit that alters electrophysiological characteristics of the 5-HT_3_AB receptors [86], there has been an interest in targeting specific 5-HT_3_ receptor subunits (Table 1). Despite this interest, current progress in this area has been limited in part due to the difficulties involved in selectively targeting the active site of heteromeric receptors. Specific allosteric modulators and improved characterization of the receptor heteromers provide some hope that personalized approaches will eventuate. Future refinement of ligand design potentially can lead to drugs that target homomeric or heteromeric receptors with a degree of specificity that will enable targeted approaches for patients expressing specific polymorphisms (e.g., subunit B variants associated with bipolar affective disorder [49,79], Table 1) or those who have alternate loads of the different 5-HT_3_ receptor subunits (e.g., patients expressing more C or E subunits).

## 5. Current Clinically Used 5-HT_3_ Receptor-Based Therapies

**Figure 3:**
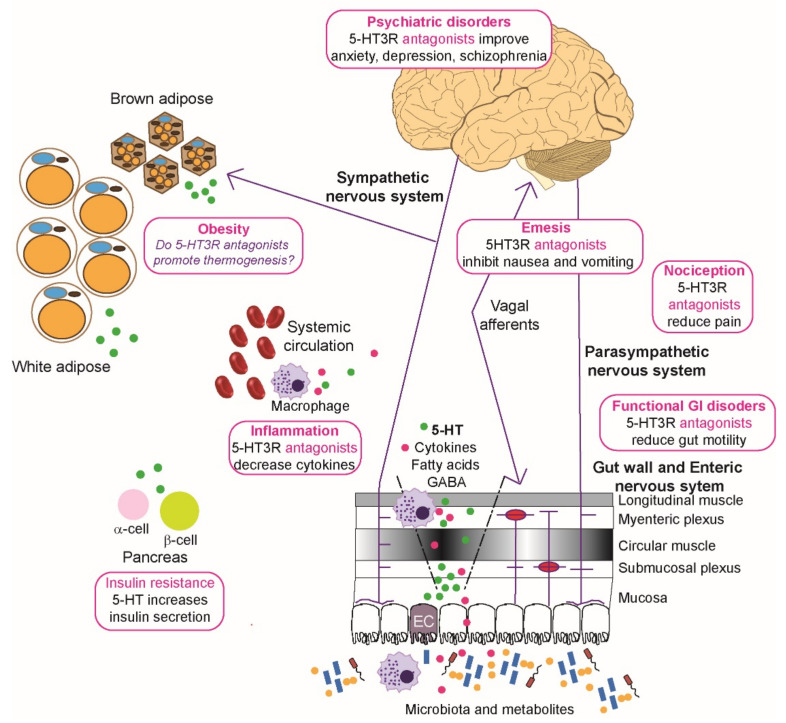
Roles of 5-HT_3_ receptors in diseases and metabolic disorders. Microbiota and their metabolites present in the lumen of the gut can affect the epithelial cells and any dendritic cells to modulate local immune function and induce enterochromaffin (EC) cells to secrete serotonin (5-HT, shown as green circles) that enters the circulation. Visceral stimulation via the sympathetic nervous system induces production of serotonin by EC cells. The enteric nervous system, consisting of submucosal plexus underneath the epithelial cells lining, interacts directly with layers of the gut wall and parasympathetic, spinal, and vagal nerves to enable bidirectional communication with the central nervous system. 5-HT release from the ECs activates 5-HT_3_ receptors on enteric neurons, vagal and spinal afferents, and these in turn relay input to the dorsal root ganglia and the brain stem. Overactivation of the chemoreceptor complex in the brainstem can lead to emesis and the processes also contribute to functional gastrointestinal (GI) disorders, such as irritable bowel syndrome, and nociception. Neuropsychiatric disorders like depression and schizophrenia are affected by disturbed 5-HT_3_ receptor signaling in the limbic region of the brain. Increased circulatory 5-HT is associated with increases in production of cytokines (shown as magenta circles), and sometimes nociception. In addition, these circulatory changes in 5-HT can potentially modify adipose function where brown adipose cells decrease thermogenesis normally raised by sympathetic activation, although this has mainly been studied using rodent models. By activating 5-HT_2_ and 5-HT_3_ receptors, 5-HT in the pancreas enhances ß cells to secrete more insulin, which acts on α cells to inhibit glucagon secretion. Physiological levels of 5-HT released by ß cells in response to glucose also lead to decrease in glucagon secretion and this effect is mediated by 5-HT_1_ receptor on α cells. For further details see the text (Section 5 and Section 6). Image drawn in Adobe Illustrator with brain image created by Hugh Guiney (CC BY-SA 3.0 https://creativecommons.org/licenses/by-sa/3.0, accessed on 25 October 2021), via Wikimedia Commons (https://commons.wikimedia.org/wiki/File:Human-brain.SVG, accessed on 25 October 2021) and red blood cells (https://commons.wikimedia.org/wiki/File:Blausen_0761_RedBloodCells.png, accessed on 25 October 2021) created by [248].

As noted above, both 5-HT_3_ receptor small molecule antagonists and agonists have been identified, but currently 5-HT_3_ receptor antagonists dominate since 5-HT_3_ receptor agonists have major emetogenic effects handicapping their clinical use (Figure 3). 5-HT_3_ receptor antagonists were initially established for clinical use to treat chemotherapy-induced nausea and vomiting (CINV) in the 1980s as ondansetron outshone the commonly used antiemetic, metoclopramide [213,214,215]. Ondansetron is still a common component in chemotherapy-induced nausea and vomiting therapies [22,30,116,216]. The evolutionary development of this 5-HT_3_ receptor antagonist class has generated many structural analogs designed to improve selectivity, reduce side effects, and improve ligand pharmacodynamics for delayed emesis compared to acute emesis, bringing additional ‘setron’ ligands, such as granisetron, ramosetron, dolasetron, tropisetron, and the second-generation ligand, palonosetron. These advances have been thoroughly reviewed [8,19,21,22,151,217,218,219]. This drug class possesses excellent antiemetic treatment and prophylaxis effects due to their ability to inhibit serotonin activity on both the central nervous system in the chemoreceptor trigger zone and peripherally on gastrointestinal vagal nerve terminals [22,30,217,218,220]. Over time, their clinical usage has expanded beyond chemotherapy induced emesis to nausea and vomiting provoked by radiation therapy and post-operative procedures in adult and pediatric patients [30,116,136,217] and also pregnancy induced emesis [217].

5-HT and its interaction with multiple types of 5-HT receptors are a critical component of the healthy functional gastrointestinal tract (Figure 3). Increases in intestinal and serum 5-HT levels are associated with the functional disorder, irritable bowel syndrome (IBS) [135,221]. 5-HT_3_ receptor antagonists (e.g., ondansetron, alosetron) are effective at treating motility problems and abdominal pain associated with diarrhea predominant irritable bowel syndrome but can lead to potential constipation [222]. Since 5-HT levels rise in diarrhea conditions, a new rationale involving use of 5-HT_3_ receptor partial agonists has been suggested. Selective partial 5-HT_3_ receptor agonists (e.g., CSTI-300) compete with high levels of endogenous 5-HT but do not completely inhibit the receptor and pre-clinical studies in animal models did not evoke constipation or emesis [184]. Therapies involving CSTI-300 or its analogs are also likely to be beneficial for patients suffering chronic diarrhea and increased circulating 5-HT such as occurs in carcinoid syndrome or diabetes induced diarrhea currently treated with 5-HT_3_ receptor antagonists [184,223,224,225].

The association of irritable bowel disease with depression and a central nervous component may in part contribute to the effectiveness of 5-HT_3_ receptor antagonists in treating the diarrhea predominant version [18,226,227,228,229,230]. The relatively few adverse effects of 5-HT_3_ receptor antagonists combined with their efficacy for treating conditions with a central component prompted investigations into their use in neurological and psychiatric disorders and are extensively reviewed [18,231,232]. 5-HT_3_ receptor antagonists show most promise in adjunct therapy for treating schizophrenia, perhaps reflective of the 5-HT_3_ receptor antagonist activity of clozapine [233,234]. Patients with schizophrenia receiving ondansetron in addition to their primary treatment of either haloperidol or risperidone showed improvement of their primary symptoms [232,235,236]. Other neurological conditions, such as obsessive compulsive disorder, may benefit from 5-HT_3_ receptor antagonist therapy but this requires further exploration [237].

5-HT_3_ receptor antagonists are also useful in treating alcohol dependence [22,41,238,239,240] but show little clinical usefulness in treating dependencies on other drugs of abuse [18,241]. Since 5-HT_3_ receptors are present in the reward center [242], this differential is unexpected. One possible contributing factor to the improved effect of 5-HT_3_ receptor antagonists on alcohol dependence may be due to ethanol acting as an allosteric modulator of 5-HT_3_ receptors [19,151,243,244,245,246,247].

Since 5-HT is an effective nociceptive inflammatory mediator, interactions between the different receptor classes are complex and this is further complicated by 5-HT_3_ receptors having a mixed nociceptive responses [249]. Both nociceptive events contributing to chronic pain and antinociceptive effects are mediated by 5-HT_3_ receptors and influence neuropathic pain in particular [249,250,251]. 5-HT_3_ receptor antagonists are used in some clinical situations to alleviate chronic pain like fibromyalgia [21,252,253]. The role of 5-HT_3_ receptor antagonists in the treatment of headaches is more controversial, with some studies reporting efficacy and others recommending against their use [254,255], although they may have roles in combatting nausea and emesis associated with some headaches. Irritable bowel syndrome is associated with abdominal pain and 5-HT_3_ receptor antagonists moderate the pain relative to placebo [256].

The efficacy of 5-HT_3_ receptor ligands is complicated by the natural heterogeneity of 5-HT_3_ receptor complexes and various polymorphisms, which may account for discrepancies in patient responses to treatments. Multiple genomic studies have revealed differences in responses to 5-HT_3_ ligands and/or clinical disorders that can be related to polymorphisms in individual subunits. Importantly, these findings indicate that there are subtle contributions of the different subunits that can have impacts on function so that, when disturbed via polymorphisms, can lead to different outcomes. For instance, single nucleotide polymorphisms in genes encoding A and B subunits are associated with bipolar affective disorder involving modifications of receptor expression and electrical properties [49,86,257,258]. Relationships between function or disorder and polymorphisms in genes encoding 5-HT_3_ receptor subunits were previously reviewed [19,21]. Advances since these reviews have continued the strong themes involving different subunits associated with clinical conditions (Table 1) highlighting the potential to target 5-HT_3_ receptors in neurological and psychiatric disorders as discussed by Fakhouri et al. [18]. Studies of considerable statistical power are required to unlock several of these associations as many polymorphisms are relatively rare and confounding interactions exist. Meta-analyses of separate studies have revealed associations that were previously obscured [44,99], enhancing our understanding of effects of polymorphisms on receptor function that will continue as genomic data becomes a more routine aspect of medicine.

## 6. 5-HT_3_ Receptors in Whole Body Metabolism

It should be kept in mind that 5-HT activates several serotonin receptors and there are recent reviews capturing interactions between 5-HT receptors in metabolism [259,260]. This review focuses on the roles of 5-HT_3_ receptors in energy homeostasis that are being unmasked. Several early studies focused on the interaction between glucose metabolism and centrally located 5-HT_3_ receptors in the brain. The effects of 5-HT_3_ receptors on peripheral glucose metabolism have been the subject of some controversy. Early findings using 5-HT_3_ receptor agonists, such as 2-methyl-5-HT, indicated that 5-HT_3_ receptors were not involved in glucose metabolism as no change in glucose, glucagon or insulin levels were detected [261]. However, administration of the 5-HT_3_ receptor agonist m-CPBG to rat brains raised blood glucose levels that could be blocked by ondansetron [262], implicating 5-HT_3_ receptors in centrally regulated whole body glucose metabolism. Now a consensus is being reached that the role of the 5-HT_3_ receptors is influenced by the metabolic state and sites of stimuli. The 5-HT_3_ receptor agonist SR-57227 that acts both centrally and peripherally [263] inhibits food intake by fasting but not fed mice [264], while in rats the effect of 5-HT_3_ receptor agonists depends on the brain region stimulated [265]. The 5-HT_2C_ receptor particularly, but also the 5-HT_1B_, 5-HT_6_ and 5-HT_3_ receptors, are all involved in regulating satiety at a central level where 5-HT itself interacts with peripheral leptin and cholecystokinin (for reviews see [8,266,267]).

In the gut, where ~95% of the body’s serotonin resides, most 5-HT is produced by a specialized subtype of endocrine cells, called enterochromaffin (EC) cells, residing alongside the epithelium lining the gut lumen. Enterochromaffin cells release 5-HT into the lamina propria, where it can signal via 5-HT receptors expressed on nerves or be inactivated by the serotonin reuptake transporter (SERT)-mediated uptake into enterocytes where it is catabolized by monoamine oxidase [268]. Indigenous spore-forming bacteria promote biosynthesis of 5-HT in enterochromaffin cells [269] and the release of 5-HT is controlled by luminal glucose status. For instance, high post-prandial glucose levels stimulate the rapid secretion of 5-HT, whereas enterochromaffin cells adapt to chronic low glucose levels by increasing transcripts of *Tph1* involved in synthesizing 5-HT [270]. The 5-HT then activates 5-HT_3_ receptors on mucosal vagal afferent terminals [271,272] (Figure 3) and this is augmented by glucose [273]. Extracellular glucose levels alter rat gastric vagal afferent 5-HT_3_ receptor density rapidly and probably by receptor trafficking as increased glucose rapidly results in more functional surface receptors [273]. Similarly, in healthy older subjects, local duodenal motor effects are modulated by 5-HT_3_ receptor mediated responses to glucose present in the small intestine [274]. However, short exposures to high fat diets compromise the ability of glucose to amplify 5-HT_3_ mediated responses in gastric vagal afferent neurons [275].

Tropisetron treatment reduced weight gain in mice with access to glucose infused water but increased their ketone body production implicating 5-HT_3_ receptors in hepatic carbohydrate and fat metabolism [276]. There also appears to be a relationship with the microbiome and inflammation and the gut brain axis with 5-HT_3_ receptors playing a role [260,277,278]. Tropisetron and palonosetron reduce endotoxin escape into the body in the *ob/ob* (leptin deficient) genetic obese model mouse and this subsequently reduces liver inflammation and fat accumulation [279]. These effects could be in part because tropisetron can reverse 5-HT inhibition of insulin release [280]. Polymorphisms in 5-HT_3_ receptor B subunits have also been related to type 2 diabetes mellitus [85], further highlighting the roles of these receptors in modulating metabolism. Exercise, which utilizes plasma glucose and fatty acids, also stimulates increases in hippocampal 5-HT levels. 5-HT_3_ receptors are critical for mediating exercise enhanced hippocampal neurogenesis, and interestingly also in the anti-depressant effects induced by exercise but not learning, as demonstrated by behavioral studies [281].

Knock out mice studies provide a powerful tool to investigate the roles of peripheral and central 5-HT in addition to the receptors mediating its action. Mice on a high fat diet are not only heavier but have increased insulin resistance and non-alcoholic fatty liver disease that can be reduced pharmacologically with inhibitors of Tph1 or genetically in mice deficient in *Tph1* [282]. Increased peripheral 5-HT is important in pregnancy to drive pancreatic beta cell expansion in mice via 5-HT_2B_ receptors [283]. Beta cells also contain 5-HT that co-locates in the vesicles with insulin [284], where it is co-secreted. 5-HT_2B_, 5-HT_1D,_ 5-HT_4_ and 5-HT_3_ receptors are found on beta cells and have autocrine responses to secreted 5-HT [259,285] regulating insulin production and glucose levels. 5-HT_3_ receptors are required for glucose-induced insulin secretion and this is more important in mice fed high fat diets where an obesity induced insulin-resistant state is developed [286] and in pregnant mice also suffering insulin resistance [287]. Both tropisetron and granisetron reduced plasma glucose levels [288,289,290], however, only tropisetron exhibited positive effects on diabetic nephropathy in rats with streptozotocin induced diabetes [288] and also lowers damage to liver tissue [289] possibly due to extra actions of tropisetron [195]. These findings implicate 5-HT_3_ receptor antagonists in improving glucose tolerance but should be treated cautiously before applying to human subjects. Ondansetron and tropisetron inhibit multidrug and toxin extrusion (MATE) and organic cation transporters (OCT) [291,292]. Although ondansetron lowered glucose levels in metformin treated healthy subjects relative to placebo, its affect alone on glucose tolerance is unclear and it is likely that pharmacodynamic interactions of the two drugs occur [293].

Polymorphisms in *Thp1* [294] and *Thp2* [295] are implicated in obesity. These findings are supported by higher *Thp1* expression in duodenum of obese subjects with an increased ability to secrete 5-HT [296]. Links between 5-HT and adipose tissue and metabolism have been reviewed [259,278,297,298] where mouse studies have been particularly informative. White adipose tissue is the major site of lipid storage whereas brown adipose tissue is involved in whole body thermogenic regulation as it contains mitochondrial uncoupling protein 1 (UCP1). In rodents, brown adipose tissue is sympathetically innervated [299,300,301,302], while it is only recently that a nerve has been associated with brown-like adipose tissue deposits in humans [303]. High fat diet induced obesity is reduced by inhibiting Thp1 or in *Thp1* deficient mice and this correlates with activation of uncoupling protein 1 (UCP1) mediated thermogenesis [282]. Mice on high fat diets have increased 5-HT levels that promote lipogenesis in white adipose tissue by activating 5-HT_2_ receptors while augmenting the suppression of thermogenesis in brown adipocytes by activating 5-HT_3_ receptors [304]. Central thermoregulatory neural circuits activating serotonergic neurons have been further implicated using fiber photometry and electrophysiology studies. Body weight and energy expenditure are modified through sympathetic regulation of brown adipose tissue that is directed through neurons in the dorsal raphe nucleus expressing melanocortin 4 receptors that in turn innervate 5-HT neurons [305]. A high fat diet is also associated with anxiety and depression that correlates with desensitization of GABAergic AgRP neurons projecting onto melanocortin 4 receptor neurons in the dorsal bed of nucleus of the stria terminus containing α5-GABA_A_ receptors and afferent serotonergic neurons with 5-HT_3_ receptors [306]. Pharmacologically or genetically subduing 5-HT_3_ receptors or enhancing α5-GABA_A_ receptors suppressed food intake and removed the high fat diet induced anxiety or depression [306]. In fact, altered food intake preferences favoring a low fat diet were seen in combined pharmacotherapies of granisetron and zonisamide [306]. Zonisamide is a sulfonamide anticonvulsant that has been considered as a clinical treatment for obesity [307,308] but not in combination with 5-HT_3_ receptor antagonists. It should be noted that 5-HT_3_ receptors are expressed widely in epileptogenic neural networks and 5-HT_3_ receptor antagonists have been associated with seizures (for a discussion see [18]), so this combination of pharmacotherapy may be effective. However, the 5-HT_3_ receptor antagonist ondansetron reduces meal evoked satiety in subjects taking a satiation nutrient drink test [309], highlighting influences of central and peripheral 5-HT_3_ receptors. Yet at this stage, links between 5-HT_3_ receptors and poor metabolic control are not properly established although some tenuous connections are indicated in Figure 3 based on rodent and human studies as discussed above.

## 7. 5-HT_3_ Receptors in Inflammation

5-HT has long been associated with inflammatory responses in part because of the extensive release of 5-HT from platelet stores upon platelet activation and stimulation of 5-HT release from enterochromaffin cells during inflammatory bowel conditions [135,310,311,312]. In addition, pro-inflammatory white blood cells, such as mast and T cells, can synthesize and selectively release 5-HT [313,314,315,316,317]. 5-HT receptors are expressed by many inflammatory cells including monocytes, macrophages, dendritic cells, T cells, B cells and mast cells (reviewed by [310,311,312]). In fact, monocytes contain several types of 5-HT receptors [311,318,319] and 5-HT modifies macrophage polarization principally via 5-HT_2B_ and 5-HT_7_ receptors [320].

Initially, a role for 5-HT_3_ receptors in regulating lymphocyte ion currents was implicated in a patch clamp study using 5-HT and the 5-HT_3_ receptor agonist 2-methyl-5-HT, where the inactivation of lymphocyte potassium ion conductance was inhibited by tropisetron (ICS-205-930) [321]. Sodium ion influx into human lymphocytes was stimulated by 5-HT_3_ receptor specific agonists further confirming the presence of 5-HT_3_ receptors [123]. However, studies into the effects of 5-HT_3_ receptors in inflammatory conditions have been confounded by various off-target effects of the commonly used 5-HT_3_ receptor antagonists such as tropiseton [38,194,195]. Tropisetron has been implicated in modulating calcineurin regulated nuclear factor of activated T cells pathways independently of 5-HT_3_ receptors [18,322,323] which is of interest as calcineurin is likely to affect sensitivity of 5-HT_3_ receptors to 5-HT [172]. Studies using cell lines have also been complicated by the presence of 5-HT in serum [311,315].

A complex interplay is likely to exist between signaling mediated by toll-like receptors (TLRs) recognizing molecules associated with microbial infection, such as lipopolysaccharide, and 5-HT receptors. For instance, TLR2 and TLR4 knock out studies in mice have shown changes in 5-HT_2_, 5-HT_3_, 5-HT_4_ and 5-HT_7_ receptor transcript expression and, importantly, this correlates with changes in function in the ileum and colon [324,325]. In addition, 5-HT modulates the lipopolysaccharide-induced release of various pro-inflammatory cytokines such as interleukin-1β (IL-1β), IL-6, IL-8/CXCL8, IL-12p40, tumor necrosis factor α (TNFα), but not IL-18 and interferon γ (IFNγ), from human monocytes [318]. A 5-HT_3_ receptor knockout study reveals its role in mediating IL-6 production and intestinal mobility contributing to rotavirus-induced diarrhea [326].

Expression levels of 5-HT_3_A receptors are increased in asthmatic patients or those exposed to air pollution [128] suggesting a response to immune challenge. The activation of 5-HT_3_ receptors forms part of the allergen-induced inflammation responses in mast cells within nodose ganglia [327]. Further evidence for a role for 5-HT_3_ receptors comes from a study showing that tropisetron dose dependently inhibited lipopolysaccharide-induced TNFα and IL-1β secretion in human monocytes [121,124]. However, neither mRNA levels of these cytokines nor the transcriptional activity of TNFα promoter were affected by tropisetron in lipopolysaccharide-activated human peripheral monocytes. Instead, the pro-inflammatory cytokines are selectively inhibited at post-translational level, and the anti-inflammatory effects of tropisetron involve inhibition of p38 mitogen-associated protein kinase [328].

Several other immune related cells contain functional 5-HT_3_ receptors. For instance, dolasetron was found to be the more effective than tropisetron or granisetron in reducing prostaglandins and IL-6 secreted by human primary chondrocytes induced by IL-1β [329]. Alveolar epithelial type II cells are regulators of immune function in the lung and contain several functional 5-HT receptors including 5-HT_3_ receptors that stimulate release of IL-8 [330]. Ondansetron, palonosetron, and ramosetron blocked A549 human lung adenocarcinoma cell proliferation in a dose-dependent manner and promoted autophagy via the extracellular signal-regulated kinase pathway [331], indicating the anti-tumor potential of 5-HT_3_ receptor antagonists. Similarly, dendritic cells also contain several 5-HT receptors including 5-HT_3_ receptors that stimulate release of pro-inflammatory cytokines [332,333]. Interestingly, dendritic cells matured in the presence of 5-HT switch to secreting the anti-inflammatory cytokine, IL-10 [333]. Notably, IL-10 can reprogram macrophage metabolism by enhancing selective degradation of mitochondria via autophagy (mitophagy), thus preventing accumulation of dysfunctional and ROS-producing mitochondria, promoting macrophages with fewer dysfunctional mitochondria [334].

Macrophages will contribute to any inflammation present in functional digestive tract disorders where 5-HT_3_ receptors are the target, in part because they are widely found in the enteric nervous system. Various studies have revealed that the microbiome alters with inflammatory bowel conditions [277,278,298] and this is probably associated with influx of host macrophages. Macrophages in the small intestine lamina propria express the greatest proportion of 5-HT_3_ receptors [335,336] and peritoneal macrophages express 5-HT_3_ receptors in mouse models [337]. A mouse model of post-operative ileus supports the use of 5-HT_3_ receptor antagonists to reduce inflammation by preferentially targeting peritoneal macrophages expressing 5-HT_3_ receptors [337]. 5-HT upregulates pro-inflammatory mediators (e.g., inducible nitric oxide synthase (iNOS), TNFα, IFNγ and IL-17A) via 5-HT_3_ receptors as this was inhibited by ondansetron or ramosetron in a dextran sulfate sodium-induced colitis mouse model [335]. Post-inflammatory visceral hypersensitivity in male rats that had recovered from acetic acid-induced colitis was relieved by 5-HT_3_ receptor antagonists, alosetron and granisetron [338]. Granisetron also alleviates colonic levels of cytokines and histological inflammatory appearance in acetic acid-induced colitis [339].

5-HT_3_ receptor antagonists thus have the potential to suppress pro-inflammatory cytokine production and so reduce inflammation. No studies have investigated how the different subunits contribute to 5-HT_3_ receptor function in immune cells. Microarray studies suggested that only very low levels of genes encoding 5-HT_3_ receptor subunits were found in white blood cells [127] despite the several lines of evidence for receptor function discussed above. More recent RNAseq studies on human lymphoblastoid cell lines have revealed that the A, B and C subunit transcripts are all equally distributed [23,340]. This finding suggests that 5-HT_3_ receptors containing A homomers, and/or AB or AC heteromers are likely to be present in white blood cells.

Rats with diabetes induced by streptozotocin and treated with tropisetron had decreased levels of oxidative stress and TNFα as well as reduced urinary cytokine secretion [288] and showed renoprotective effects in early-stage diabetic nephropathy by blocking calcineurin/nuclear factor of activated T-cell pathway [322]. This pathway was inhibited by tropisetron to halt the antigen-induced proliferation of human peripheral T cells and the generation of IL-2 [323]. In a recent cohort study, ondansetron usage was associated with reduced risk-adjusted in-hospital mortality in critically ill patients suffering from acute kidney injury [341]. Anti-inflammatory effects of 5-HT_3_ receptor antagonists underscore the potential of 5-HT_3_ receptors as immunomodulatory targets in autoimmune diseases, including multiple sclerosis and rheumatoid arthritis [310].

## 8. Conclusions

Although 5-HT_3_ receptors play roles in the regulation of metabolism and inflammation, further studies are required to elucidate the complex interplay of the large number of functional 5-HT_3_ receptors, their contribution to energy homeostasis, and their detailed biochemical and pharmacological properties. Despite recent advances in the field, the comprehension of 5-HT_3_ receptor physiological functions is hindered due to complex expression patterns, numerous isoforms, and subunit types. This is further complicated by difficulties in studying different types of heteromers, whose assembly, composition, stoichiometry and expression in diverse cell types and tissues appears to have significant physiological implications but is poorly understood. The function of most available drugs has been studied using 5-HT_3_A homomeric receptors and, to a lesser extent, the AB heteromer. However, rare genetic variations in genes encoding other 5-HT_3_ receptor subunits also contribute to a number of disorders (Table 1). Therefore, a thorough characterization of heteromeric receptors and determination of their pharmacology is required, as subunit arrangements influence ligand binding kinetics and the subsequent physiological function of the receptor. Further challenges are imposed by the fact that rodents lack the 5-HT_3_ receptor C, D and E subunits, complicating the practicality of in vivo genetic studies. Finally, although most attention has been focused on 5-HT_3_ receptors present at the cell surface, the receptors are also distributed on intracellular organelles. However, the roles and subunit composition of organelle-located 5-HT_3_ receptors are still to be investigated. Interestingly, 5-HT_3_ receptors are present in mitochondrial membranes, where they augment calcium ion uptake in hypoxia and increase the respiration control ratio in mice [342]. Furthermore, emerging studies revealed that blood in the normal physiological state contains whole functional cell-free mitochondria [343,344]. This observation may have profound implications for the field of inflammation and clinical applications [343], potentially involving signaling via 5-HT_3_ receptors present in these mitochondrial membranes.

In conclusion, emerging studies suggest there is much potential for therapeutic intervention in areas beyond those for which 5-HT_3_ receptors are currently used. Collectively, genetic animal models combined with the identification of genetic variants and the increasing availability of human genotyping will be of benefit in gaining insight into metabolic signaling. Targeting 5-HT_3_ receptors may form a new prospect for the personalized treatment of immune and metabolic diseases.

## Figures and Tables

**Table 1:** Summary of disorders related to mutations of *HTR3* genes encoding 5-HT_3_ receptor subunits ^1^.

Gene	SNP ^2^ Number	Phenotype and Condition Affected	Reference
*HTR3A*	rs1150226	Temporal lobe activity	[39]
		Substance dependence (alcohol, nicotine)	[40,41]
	rs1062613	Irritable bowel syndrome (IBS)	[42,43,44]
		Dyspepsia and visceral symptoms	[45]
		Pain and fibromyalgia syndrome	[46,47]
		Bipolar affective disorder ^3^	[48,49,50]
		Depression and response to paroxetine	[51,52,53,54]
		Schizophrenia and treatment response ^3^	[55,56,57,58,59]
		Substance dependence (nicotine) ^3^	[40,60]
		Disorders associated with childhood abuse	[50,54,61,62]
	rs1150220	Autism	[63]
	rs10160548	Substance dependence (alcohol, nicotine)	[40,60,64]
	rs1176713	Schizophrenia and risperidone response	[65]
		Substance dependence and ondansetron	[41,66]
		Post-operative vomiting	[67]
		Depression and bipolar disorder	[68,69]
	rs1176719	Bipolar affective disorder	[68]
*HTR3B*	rs3758987	Substance dependence (heroin, nicotine)	[60,70]
		Obsessive compulsive disorder	[71]
		Post-operative vomiting	[72]
	rs3831455	CINV and paroxetine induced nausea with alterations in responses to ondansetron and ramosetron	[73,74,75,76]
		Treatment resistant schizophrenia	[77]
	rs1176744	Major depression and bipolar affective disorder	[49,78,79]
		Nausea and vomiting (CINV, POV) ^3^	[74,75,76,80,81]
		Eating disorders and obsessive compulsive disorder	[71,82]
		Substance dependence (alcohol, nicotine)	[60,64,83]
		Pain	[46,84]
		Diabetes mellitus type 2 (DM2)	[85]
		Differences in channel characteristics and drug efficacy (paroxetine, setrons, topotecan)	[76,80,86,87,88,89]
	rs3782025	Substance abuse (alcohol)	[64,66]
		CINV involving opioid treatment	[81]
		Attention deficit/hyperactivity disorder	[90]
	rs1672717	Substance abuse (nicotine)	[60]
		CINV involving opioid treatment	[81]
		Attention deficit/hyperactivity disorder	[90]
	rs12795805	Diabetes mellitus type 2 (DM2)	[85]
*HTR3C*	rs6766410	CINV ^3^	[91,92,93,94]
		Autism and obsessive compulsive disorder	[71,95,96]
		Ondansetron effect in diarrhea predominant IBS	[97]
	rs6807362	CINV and pregnancy induced nausea	[92,98,99]
		Autism	[95]
	rs6807670	Pregnancy induced nausea	[98]
*HTR3D*	rs6443930	CINV and lack of therapeutic response	[100]
		Obsessive compulsive disorder	[71]
	rs12493550	Primary angle-closure glaucoma	[101]
*HTR3E*	rs56109847	Diarrhea predominant IBS	[42,44]
	rs7627615	Schizophrenia	[102,103]
		Obsessive compulsive disorder ^3^	[96,104]

^1^ For further details in relation to studies before 2011 refer to [19,21]. ^2^ Abbreviations: CINV—chemotherapy induced nausea and vomiting; DM2—Diabetes mellitus type 2; IBS—irritable bowel syndrome; POV—post-operative vomiting; SNP—single nucleotide polymorphism. ^3^ Reports have contradictory findings.

## Data Availability

No new data were created or analyzed in this study. Data sharing is not applicable to this article.
